# What does Weber’s law tell us about spike statistics?

**DOI:** 10.1186/1471-2202-13-S1-P136

**Published:** 2012-07-16

**Authors:** Harel Z Shouval, Animesh Agarwal, Jeff Gavornik

**Affiliations:** 1 Department of Neurobiology and Anatomy, University of Texas Medical School at Houston, Houston, TX, 77030, USA; 2 Howard Hughes Medical Institute, the Picower Institute of Learning and Memory, Massachusetts Institute of Technology, Cambridge, MA 02142, USA

## 

**Figure 1:**
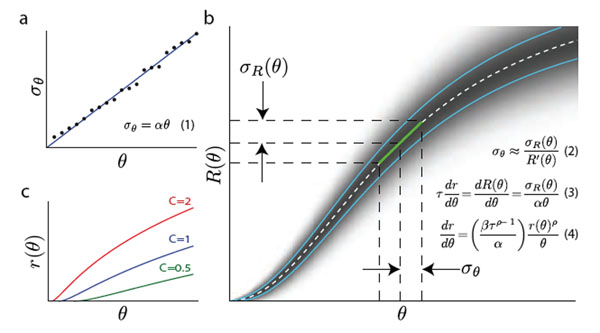
Weber’s law and Neuronal Statistics. **a.** Weber’s law: estimate errors σ_θ_ increase linearly with stimulus intensity θ. **b**. General scheme and derivation of differential equation **c.** example tuning curves with Poisson spike statistics for different values of the integration constant. The *log-power* curves for different integration constants, have the form:

Weber's law (Fig. 1A), which states that estimation error scales linearly with the magnitude of the estimated variable, is a ubiquitous and robust phenomenon. A similar observation in estimating temporal intervals is called Scalar Timing. A central goal of neuroscience is to find the physiological basis of behavior; yet, despite the fundamental nature of Weber's law and its long history, its physiological basis remains unknown. This work poses a simple question- what are the statistical characteristics of spiking neural-processes that can lead to Weber’s law? Our analysis is based on the hypothesis that neuronal noise, or more precisely the variability of neural spike counts, is the origin of perceptual errors.

We develop a simple differential equation, based on a local linear approximation, that relates perceptual performance to neural spike statistics. This equation has the form:

where r(θ) is the form of the tuning curve, α the Weber fraction, τ the time window over which spike statistics are sampled and the parameters ρ and β characterize the spike count statistics. For example for Poisson statistics ρ=0.5 and β=1. This equation has an analytical solution of the form:

where K=(±β(1-ρ)/α)^n^_/_τ and n=1/(1-ρ). Figure 1B below shows parts of the derivation, and Figure 1C examples of log-power tuning curves. All the parameters of this function except one are determined by the spike statistics, and the free parameter does not alter the scaling. More complex exact calculations confirm the validity of the results obtained using the linear approximation. We also compare our predictions to available data.

This analysis can also be applied in the temporal domain to the scalar timing law using spike rates that vary in time with a *log-power* profile. Our linear approximation demonstrates the principles of our theory and a discrete series approximation captures almost exactly the results of stochastic simulations.

We also test the optimality of these scaling laws using use variational calculus to find a joint differential equation for the tuning curves and the probability distribution of the measured variable. In a surprisingly general result, this analysis shows that Weber’s law is optimal only if the estimated variable has a scale invariant power-law distribution with an exponent of *n*=*-2*.

